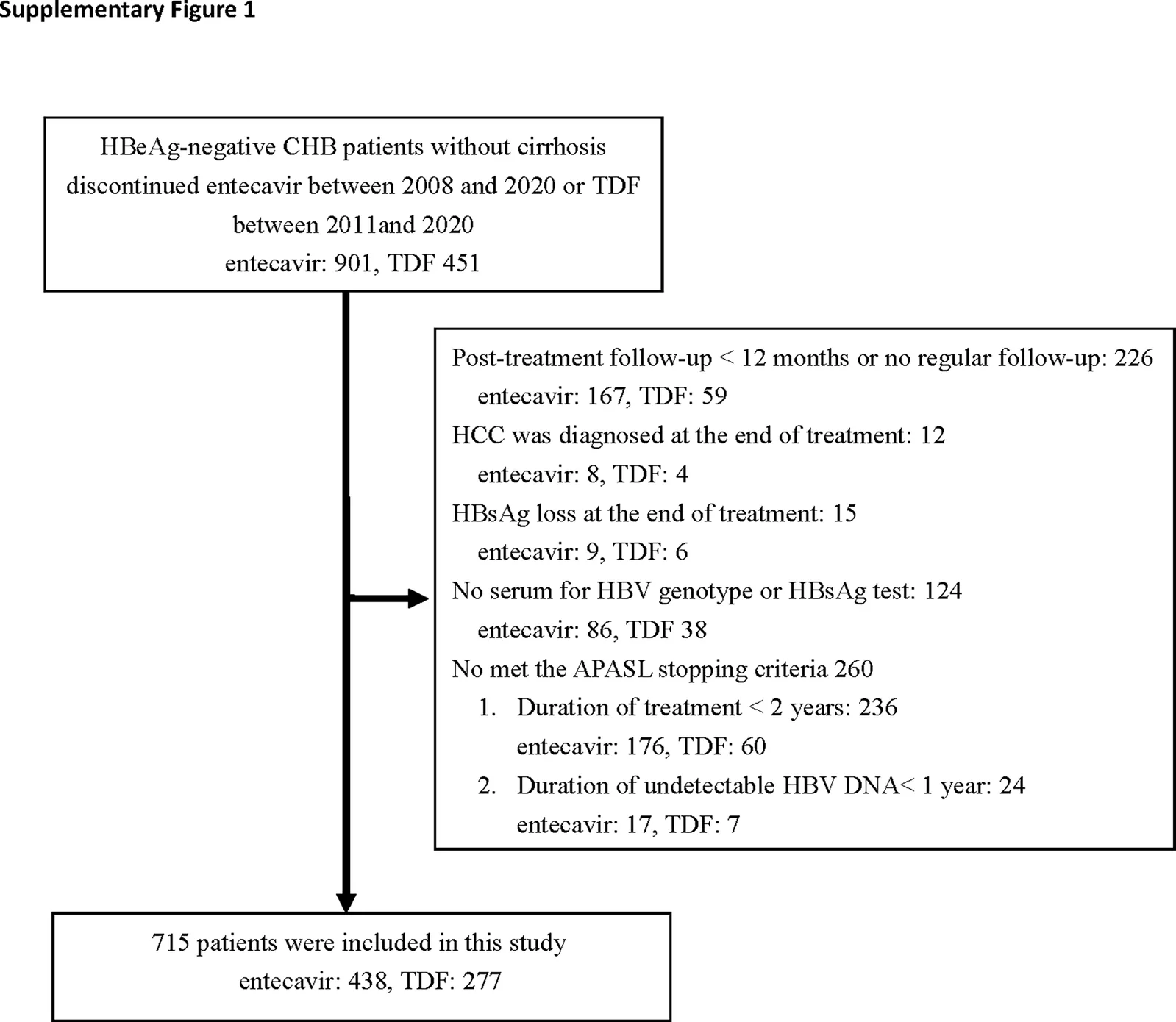# qHBsAg outperforms ALT/qHBsAg ratio in predicting HBsAg seroclearance after cessation of entecavir versus tenofovir

**DOI:** 10.1016/j.bj.2026.100945

**Published:** 2026-01-13

**Authors:** Hsing-Yun Lee, Cheng-Yuan Peng, Kwong-Ming Kee, Tsung-Hui Hu, Jing-Houng Wang, Chao-Hung Hung, Sheng-Nan Lu, Chien-Hung Chen

**Affiliations:** a Division of Hepatogastroenterology, Department of Internal Medicine, Kaohsiung Chang Gung Memorial Hospital and Chang Gung University College of Medicine, Kaohsiung, Taiwan; b Center for Digestive Medicine, Department of Internal Medicine, China Medical University Hospital, Taichung, Taiwan; c School of Medicine, China Medical University, Taichung, Taiwan

**Keywords:** Entecavir, Tenofovir, HBsAg, **A**kaike information criterion, Bayesian information criterion

## Abstract

**Background:**

We aimed to establish the optimal value of the alanine aminotransferase (ALT)/quantitative HBsAg (qHBsAg) ratio and qHBsAg level at end of treatment (EOT) to predict HBsAg seroclearance following cessation of entecavir versus tenofovir disoproxil fumarate (TDF) in HBeAg-negative with chronic hepatitis B (CHB) patients.

**Material and methods:**

This retrospective cohort study included 715 HBeAg-negative patients without cirrhosis who had previously received entecavir (*n* = 438) or TDF (*n* = 277).

**Results:**

The cumulative incidences of HBsAg seroclearance at 5 and 10 years after cessation of entecavir or TDF were 13.0 % and 33.8 %, respectively. Both a qHBsAg level <100 IU/mL and an ALT/qHBsAg ratio ≥0.2 at EOT were independent predictors of HBsAg seroclearance (*p* < 0.001). A qHBsAg of 100 IU/mL had significantly lower Akaike information criterion (AIC) and Bayesian information criterion (BIC) values than an ALT/qHBsAg ratio of 0.2 at EOT (AIC: 1273 vs. 1305, BIC: 1276 vs. 1308, both *p* < 0.001). Additionally, patients who discontinued entecavir therapy had a higher rate of HBsAg seroclearance than patients who discontinued TDF therapy, regardless of qHBsAg ≥100 IU/mL (*p* = 0.027) or ALT/qHBsAg ratio <0.2 (*p =* 0.028). The optimal values for predicting HBsAg seroclearance were 300 and 100 IU/mL for qHBsAg and 0.1 and 0.2 for the ALT/qHBsAg ratio after stopping entecavir versus TDF, respectively.

**Conclusions:**

A qHBsAg level <100 IU/mL at EOT is better predictor of HBsAg seroclearance than an ALT/qHBsAg ratio ≥0.2. Compared to TDF, entecavir was associated with higher rates of HBsAg seroclearance in patients with higher qHBsAg level or a lower ALT/qHBsAg ratio at EOT.

## Highlights

• qHBsAg level <100 IU/mL is better predictor of HBsAg loss than ALT/qHBsAg ratio ≥0.2.
• Entecavir had a higher HBsAg loss rate compared to TDF in patients with higher qHBsAg level or lower ALT/qHBsAg ratio.
• Entecavir have a higher potential for achieving HBsAg loss than TDF.

## 1 Introduction

Chronic hepatitis B (CHB) is one of the leading causes of liver disease worldwide and was responsible for an estimated 1.1 million deaths in 2022 [1]. Nucleos(t)ide analogues (NAs) have been proven to improve the clinical outcomes of patients with CHB and are widely used [2,3]. However, NAs rarely achieve a functional cure due to their limited effect on covalently closed circular DNA (cccDNA), the key driver of viral persistence. Thus, long-term use of NAs is typically necessary [4].

Currently, there are differing opinions on the NA discontinuation criteria for patients with HBeAg-negative CHB [[5], [6], [7]]. Although discontinuing NAs may result in a high risk of HBV relapse, it also increases the likelihood of HBsAg seroclearance [[8], [9], [10], [11]]. Therefore, it is crucial to establish comprehensive criteria for treatment discontinuation in HBeAg-negative CHB patients.

Recently, a study of patients with non-cirrhotic, HBeAg-negative CHB (predominant) or HBeAg-positive CHB with undetectable HBV DNA (<20 IU/mL) at the end of treatment (EOT) treated with entecavir alone reported that an alanine aminotransferase (ALT)/quantitative HBsAg (qHBsAg) ratio ≥0.2 at EOT could predict HBsAg seroclearance [12]. This ratio reflects both host immune activity (ALT) and viral antigen burden (HBsAg), which are two major determinants of HBsAg clearance. A higher value of ALT/qHBsAg ratio value may potentially provide a more comprehensive assessment of the host–virus interaction, a condition favorable for HBsAg clearance. However, due to the small sample size and inclusion of only entecavir-treated patients, it remains unclear whether the ALT/qHBsAg ratio can reliably predict HBsAg seroclearance after discontinuation of other antiviral agents, including tenofovir disoproxil fumarate (TDF).

Many studies, including the Asian Pacific Association for the Study of the Liver (APASL) guidelines, recommend that a qHBsAg level <100 IU/mL at EOT is a suitable indicator for stopping treatment [8,[13], [14], [15], [16]]. Therefore, it is necessary to compare the ability of an ALT/qHBsAg ratio of ≥0.2 and a qHBsAg level <100 log IU/mL at EOT to predict HBsAg seroclearance after discontinuation of NA therapy. Moreover, our previous study showed that patients who discontinued TDF had significantly higher rates of HBsAg seroclearance than patients who discontinued entecavir in patients without HBV relapse after cessation of therapy [11]. Therefore, it is unclear whether cut-off values of HBsAg levels or the ALT/HBsAg ratio at EOT is different to predict HBsAg seroclearance between patients who discontinued entecavir and TDF**.** Therefore, this study will establish the cut-off values of HBsAg levels or the ALT/HBsAg ratio at EOT to predict HBsAg seroclearance after cessation of entecavir versus TDF therapy**.**

## 2 Patients and methods

### 2.1 Patients

[Supplementary Fig. 1] shows the flow chart of patients enrolled in this study. This retrospective study included 438 patients with HBeAg-negative CHB without cirrhosis who received entecavir treatment between 2008 and 2020 and 277 patients with HBeAg-negative CHB without cirrhosis who received TDF treatment between 2011 and 2020 from Kaohsiung Chang Gung memorial hospital. Moreover, 115 patients who received entecavir or TDF therapy (2008–2021) were included (entecavir, n = 87; TDF, n = 28) from China Medical University Hospital, as validation cohort. All patients were followed-up post-treatment for at least 12 months. The endpoint of follow-up was the last visit for both patients with or without retreatment.

Patients were excluded if they showed any evidence of autoimmune hepatitis, alcoholic liver disease, or markers of hepatitis C virus (HCV), hepatitis D virus (HDV), or human immunodeficiency virus. Patients were also excluded if they achieved HBsAg loss during treatment, had decompensated liver cirrhosis or HCC, or received immunosuppressive therapy.

The absence of cirrhosis at baseline was confirmed by biopsy (*n* = 26) or combined repeated ultrasound findings indicating an absence of clinical features such as splenomegaly, ascites, or gastroesophageal varices. Taiwan's National Health Plan reimburses patients with HBeAg-negative CHB without cirrhosis for 3 years of NA therapy if they meet the initial criteria for starting NA therapy. In this study, most patients (80 %) stopped either entecavir or TDF therapy according to the criteria of Taiwan's National Health Plan in order to avoid personally incurring the cost of the drugs. All patients met the stopping criteria for antiviral agents based on the 2012 guidelines of the APASL [17]. APASL guideline recommend the treatment can be withdrawal after treatment for at least 2 years with undetectable HBV DNA documented on three separate occasions, 6 months apart in HBeAg-negative patients. The stopping criteria are the same for entecavir and TDF therapy.

This study was conducted in accordance with the Declaration of Helsinki of 1975. All patients provided written informed consent and the Research Ethics Committees of Chang Gung Memorial Hospital approved the study.

### 2.2 Methods

After the cessation of either entecavir or TDF therapy, patients were followed up every 1–3 months during the first 6 months and then every 3 months until their last hospital visit. Biweekly or weekly visits were conducted if a patient's alanine aminotransferase (ALT) levels increased beyond the upper limit of normal (ULN; 40 U/L). Serum HBV DNA was assayed every 1–3 months for the first 6 months after the cessation of therapy and then every 3–6 months thereafter. HBsAg was quantified retrospectively using stored serum samples collected prior to initiation of treatment and at the EOT.

### 2.3 Definition of HBV relapse and criteria for retreatment

The definitions of virological relapse, clinical relapse, retreatment and HBsAg seroclearance are shown in [Supplementary Table 1].^17^ The duration of consolidation was calculated from the first demonstration of undetectable HBV DNA until EOT in HBeAg-negative patients. The FIB-4 index was calculated using the following formula: aspartate - aminotransferase (AST) [IU/L] × age [years]/platelet count [10^9^/L] × ALT [IU/L]^1/2^ [18].

The criteria for retreatment of patients with HBeAg-negative CHB without cirrhosis are two elevations in ALT (≥2 × ULN) at least 3 months apart, and HBV DNA ≥2000 IU/mL. The criteria for retreatment of hepatic decompensation in Taiwan's National Health Plan are total bilirubin ≥2 mg/dL or prothrombin time prolongation ≥3 seconds, regardless of HBV DNA levels.

### 2.4 Serology

Serum HBsAg, HBeAg, and anti-HCV antibodies were measured using commercially available assay kits (HBsAg EIA, HBeAg EIA, and anti-HCV EIA 3.0; all from Abbott, Chicago, IL, USA). HBsAg levels were quantified using Elecsys HBsAg II Quant reagent kits (Roche Diagnostics, Indianapolis, IN, USA) in accordance with the manufacturer's guidelines. The detection limit of the kit is 0.05 IU/mL. HBV DNA was measured using the COBAS TaqMan HBV test (CAP-CTM; Roche Molecular Systems, Inc., Branchburg, NJ, USA), which has a detection threshold of 20 IU/mL.

### 2.5 HBV genotyping

HBV genotypes were identified using a restriction fragment length polymorphism (RFLP) assay that targets the surface gene (nucleotide positions 256 to 796), following a previously established method [19]. In cases where the RFLP results were unclear or suggested mixed infection, direct sequencing of the surface gene was performed for confirmation.

### 2.6 Statistical analysis

Data are expressed as mean ± standard deviation (SD), proportion, or median (range). Categorical variables were analyzed using chi-squared tests and continuous variables were assessed with the Student's t-test. The Kaplan-Meier method and log-rank test were employed to evaluate the cumulative incidences of HBsAg loss. Cox proportional hazards regression models were used for both univariate and multivariate analyses. Variables with *p-*values <0.2 in the univariate analysis were included in the multivariate analysis. The Cox model with the forward selection method was applied to identify independent risk factors. A time-dependent receiver-operating characteristic (ROC) curve analysis was used to assess the best HBsAg cut-off level for predicting HBsAg seroclearance within 8 years after cessation of NA therapy [20]. All statistical tests were two-tailed, with significance set at *p* < 0.05. The predictive value of a qHBsAg level and an ALT/qHBsAg ratio for HBsAg seroclearance after cessation of entecavir or TDF was compared using the Akaike information criterion (AIC) and the Bayesian information criterion (BIC).

## 3 Results

### 3.1 Clinical characteristics of the study population treated with entecavir or TDF

[Table 1] summarizes the clinical characteristics of the patients with HBeAg-negative patients without cirrhosis treated with entecavir or TDF. At the start of treatment, the entecavir group had lower HBV DNA levels and a smaller proportion of NA-naive patients than the TDF group. At EOT, the entecavir group had lower ALT levels and had experienced longer durations of treatment and consolidation than the TDF group. In addition, the entecavir group had longer times to clinical relapse, retreatment, and HBsAg seroclearance compared to the TDF group.

**Table 1:** Clinical characteristics of study population treated with entecavir or TDF treatment.

	Entecavir n = 438	TDF n = 277	*p* value
Initial treatment			

Age (years)	51.7 ± 10.6	52.5 ± 10.9	0.321
Sex (male: female)	347:91	212:65	0.396
ALT (U/L)	315.0 ± 464.5	313.4 ± 450.4	0.963
Total bilirubin (mg/dL)	1.90 ± 2.86	1.79 ± 3.27	0.624
HBV DNA (log IU/mL)	5.62 ± 1.54	6.04 ± 1.48	<0.001
HBV genotype B versus C	351 versus 87	203 vs. 74	0.033
FIB-4	3.10 ± 2.76	3.09 ± 3.16	0.966
NA-naive	309 (63.4 %)	166 (67.4 %)	0.003
HBsAg (log IU/mL)	2.96 ± 0.84	2.98 ± 0.83	0.803

End of treatment			

Age (years)	54.9 ± 10.8	55.5 ± 10.8	0.427
ALT (U/L)	26.8 ± 15.8	34.1 ± 29.3	<0.001
Total bilirubin (mg/dL)	0.93 ± 0.40	1.01 ± 1.23	0.215
Treatment duration (weeks)	172.4 ± 61.6	159.5 ± 16.9	<0.001
Consolidation duration (weeks)	141.5 ± 62.6	126,5 ± 25.2	<0.001
HBsAg (log IU/mL)	2.40 ± 0.85	2.35 ± 0.82	0.401
ALT/HBsAg	2.53 ± 17.6	2.82 ± 23.7	0.850

After EOT			

Development of clinical relapse	241 (55.0 %)	165 (72.7 %)	0.232
Time to clinical relapse (weeks)	74.8 ± 65.2	49.2 ± 49.0	<0.001
Retreatment	207 (47.3 %)	142 (51.3 %)	0.297
Time to retreatment (weeks)	101.3 ± 78.6	78.6 ± 68.1	0.005
HBsAg seroclearance	85 (19.4 %)	33 (11.9 %)	0.009
Time to HBsAg seroclearance (weeks)	253.0 ± 157.6	155.0 ± 121.7	0.002

ALT, alanine aminotransferase; HBV, hepatitis B virus; HBsAg, hepatitis B surface antigen; HBeAg, hepatitis B e antigen; ETV, entecavir: NA, nucleoside analogues; TDF, *tenofovir disoproxil* fumarate.

### 3.2 Clinical characteristics of patients with and without post-treatment HBsAg seroclearance

The clinical characteristics of the patients who achieved and did not achieve HBsAg seroclearance are presented in [Table 2]. Patients who achieved HBsAg seroclearance were younger and had lower HBV DNA and qHBsAg levels at initiation of treatment and were more likely to have been treated with entecavir than the patents who did not achieve HBsAg seroclearance. At EOT, patients who achieved HBsAg seroclearance were younger, had longer durations of treatment and consolidation, and had lower qHBsAg levels and a higher ALT/qHBsAg ratio than the patients who did not achieve HBsAg seroclearance.

**Table 2:** Clinical characteristics of study population with or without HBsAg seroclearance.

	No HBsAg seroclearance n = 597	HBsAg seroclearance n = 118	*P* value
Initial treatment			

Age (years)	52.5 ± 10.8	49.5 ± 9.6	0.005
Sex (male: female)	463:134	96:22	0.361
ALT (U/L)	313.7 ± 444.6	317.5 ± 527.0	0.936
Total bilirubin (mg/dL)	1.84 ± 3.08	1.94 ± 2.75	0.733
HBV DNA (log IU/mL)	5.93 ± 1.46	5.07 ± 1.69	<0.001
HBV genotype B versus C	377 vs. 116	77 vs. 45	<0.001
FIB-4	3.10 ± 2.82	3.04 ± 3.35	0.827
Entecavir: TDF	353:244	85:33	0.009
NA-naive	403 (67.5 %)	72 (61.0 %)	0.173
HBsAg (log IU/mL)	3.04 ± 0.78	2.58 ± 1.00	<0.001

End of treatment			

Age (years)	55.5 ± 11.1	53.1 ± 9.23	0.023
ALT (U/L)	29.4 ± 22.6	30.9 ± 20.3	0.488
Total bilirubin (mg/dL)	0.97 ± 0.90	0.96 ± 0.40	0.907
Treatment duration (weeks)	164.2 ± 37.1	187.4 ± 87.6	<0.001
Consolidation duration (weeks)	130.7 ± 40.3	161.0 ± 86.1	<0.001
HBsAg (log IU/mL)	2.54 ± 0.67	1.59 ± 1.10	<0.001
ALT/HBsAg	0.53 ± 3.00	13.32 ± 4.40	<0.001

After EOT			

Retreatment	328 (54.9 %)	21 (17.8 %)	<0.001

ALT, alanine aminotransferase; HBV, hepatitis B virus; HBsAg, hepatitis B surface antigen; HBeAg, hepatitis B e antigen; ETV, entecavir: NA, nucleoside analogues; TDF, *tenofovir disoproxil* fumarate.

### 3.3 Incidences and predictors of HBV relapse, retreatment and HBsAg seroclearance after cessation of entecavir or TDF

In the total cohort, the cumulative incidences of virological and clinical relapse, retreatment and HBsAg seroclearance at 3, 5, 8 years are presented in [Supplementary Table 1]. The cumulative incidences of HBsAg seroclearance at 3, 5, and 8 years after stopping entecavir versus TDF therapy, respectively were 7.7 % versus 7.8 %, 13.4 % versus 11.7 %, and 23.2 % versus 16.7 %, respectively. There was no significant difference in the HBsAg loss rate between the entecavir and TDF groups.

Next, we analyzed the data for all enrolled patients to identify factors that could predict HBsAg seroclearance [Table 3]. When the qHBsAg level (but not the ALT/qHBsAg ratio) at EOT was included in the model, Cox regression analysis showed that younger age, HBV genotype C, NA-experienced status, a longer duration of consolidation, a lower qHBsAg level at EOT, and no retreatment were independent factors associated with HBsAg seroclearance [Table 3, model 1]. In contrast, when the ALT/qHBsAg ratio (but not the qHBsAg level) at EOT was included, a lower HBV DNA level, HBV genotype C, NA-experienced status, a longer duration of consolidation, a higher ALT/qHBsAg ratio at EOT, and no retreatment were independent factors associated with HBsAg seroclearance [Table 3, model 2].

**Table 3:** Factors predictive of HBsAg seroclearance for all patients.

Variables	Univariate analysis		aMultivariate analysis (model 1)		aMultivariate analysis (model 2)	
	HR (95 % CI)	*p* value	HR (95 % CI)	*P* value	HR (95 % CI)	*p* value
Initial treatment						

Age (per year)	0.989 (0.972–1.006)	0.194	0.974 (0.955–0.992)	0.006		
Sex (Male vs. female)	1.253 (0.789–1.992)	0.339				
TDF vs. entecavir	0.874 (0.579–1.321)	0.523				
ALT (per U/L)	1.000 (1.000–1.000)	0.933				
Total bilirubin (per mg/dL)	1.013 (0.959–1.071)	0.646				
HBV DNA (per log IU/mL)	0.723 (0.645–0.811)	<0.001			0.828 (0.736–0.832)	0.002
HBV genotype (C vs. B)	1.943 (1.339–2.819)	<0.001	1.924 (1.307–2.831)	0.001	1.538 (1.051–2.250)	0.027
FIB-4	1.020 (0.955–1.089)	0.553				
NA-naïve (yes vs. no)	0.568 (0.391–0.826)	0.003	0.603 (0.407–0.893)	0.012	0.502 (0.339–0.742)	0.001
HBsAg (per log IU/mL)	0.481 (0.390–0.592)	<0.001				

End of treatment						

Age (years)	0.994 (0.977–1.011)	0.477				
ALT (U/L)	1.005 (0.997–1.012)	0.219				
Total bilirubin (mg/dL)	0.998 (0.773–1.287)	0.986				
Treatment duration (per week)	1.007 (1.004–1.009)	<0.001				
Consolidation duration (per week)	1.007 (1.005–1.010)	<0.001	1.003 (1.001–1.006)	0.009	1.005 (1.003–1.008)	<0.001
qHBsAg (per log IU/mL)	0.257 (0.214–0.309)	<0.001	0.297 (0.245–0.361)	<0.001	N/A	
ALT/qHBsAg ratio	1.017 (1.014–1.021)	<0.001	N/A		1.016 (1.012–1.020)	<0.001
Retreatment (yes vs. no)	0.181 (0.113–0.290)	<0.001	0.409 (0.246–0.681)	0.001	0.239 (0.147–0.388)	<0.001

ALT, alanine aminotransferase; CI, confidence interval; HR, Hazard ratio; HBV, hepatitis B virus, HBsAg: hepatitis B surface antigen; NA, nucleoside analogues; N/A: Not applicable; qHBsAg (quantitative HBsAg); TDF, tenofovir disoproxil fumarate.

a Model 1: Multivariate analysis included qHBsAg, no ALT/qHBsAg at EOT. *Model 2: Multivariate analysis included ALT/qHBsAg, no qHBsAg at EOT.

### 3.4 Cumulative incidences of HBsAg seroclearance based on an ALT/qHBsAg ratio of 0.2 at EOT

[Fig. 1] shows the cumulative incidences of HBsAg seroclearance for the cohort, stratified by an ALT/qHBsAg ratio of 0.2 at EOT. Among all patients treated with entecavir [Fig. 1A], the cumulative rates of HBsAg seroclearance at 3, 5, and 8 years after EOT were 21.1 %, 36.0 %, and 50.3 %, respectively, for patients with an ALT/qHBsAg ratio ≥0.2 and 1.7 %, 5.3 %, and 13.8 %, respectively, among patients with an ALT/qHBsAg ratio <0.2. Patients with an ALT/qHBsAg ratio ≥0.2 exhibited a significantly higher HBsAg seroclearance rate than those with a ratio <0.2 (*p* < 0.001). However, patients with an ALT/qHBsAg ratio ≥0.2 exhibited a significantly higher HBsAg seroclearance rate than those with a ratio <0.2 only in patients without retreatment (*p* < 0.001), but not patient who received retreatment (*p* = 0.488). Among all patients treated with TDF [Fig. 1B], the cumulative rates of HBsAg seroclearance at 3, 5, and 8 years were 20.9 %, 30.2 %, and 44.8 %, respectively, for patients with an ALT/qHBsAg ratio ≥0.2 and 1.3 %, 3 %, and 4.3 %, respectively, for patients with an ALT/qHBsAg ratio <0.2. Patients with an ALT/qHBsAg ratio ≥0.2 exhibited a significantly higher HBsAg seroclearance rate than those with a ratio <0.2 (*p* < 0.001), including patients with or without retreatment (both, *p* < 0.001).

**Fig. 1:**
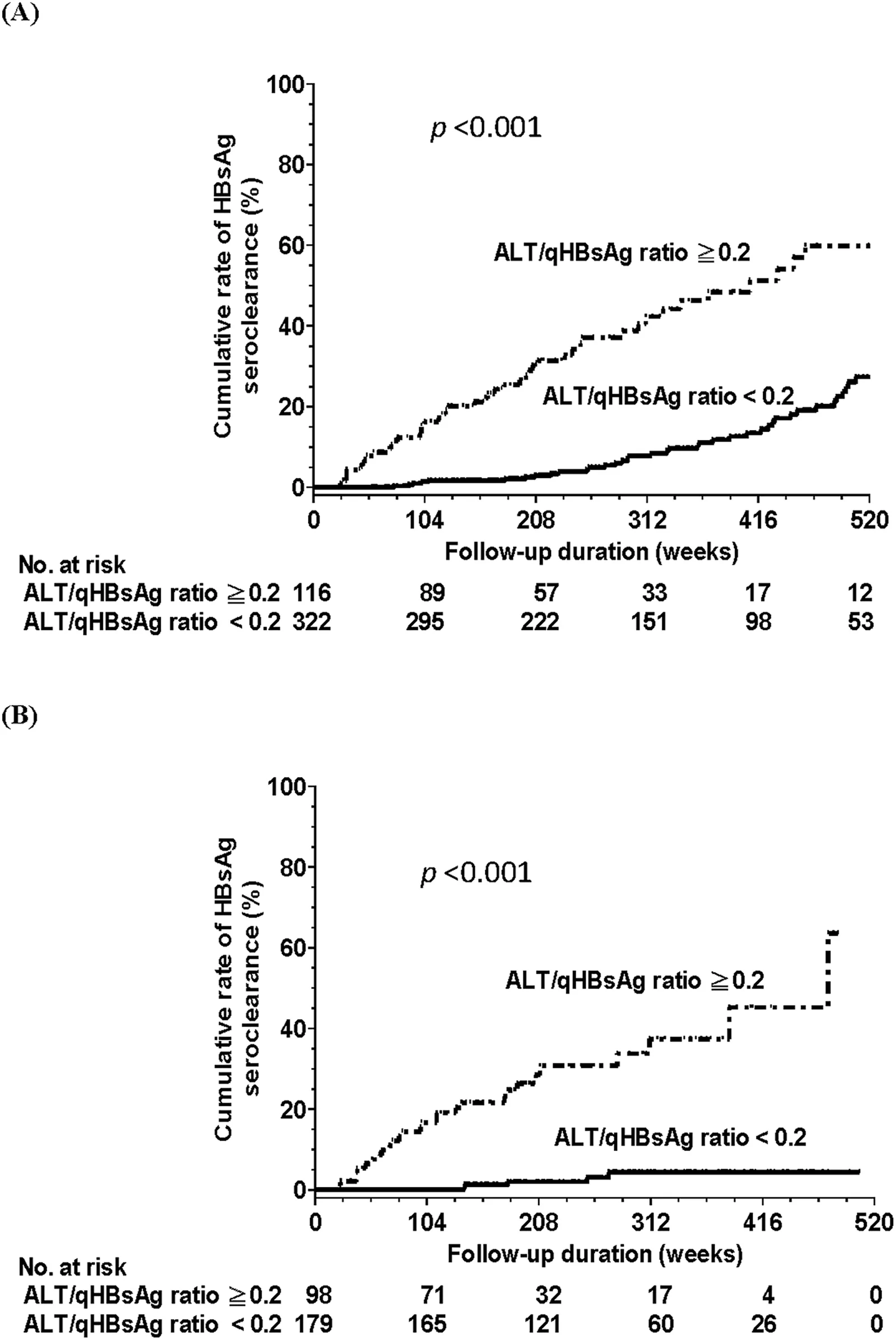
The cumulative incidences of HBsAg seroclearance in patients with HBeAg-negative chronic hepatitis B (CHB) treated with (A) entecavir or (B) *tenofovir disoproxil fumarate (TDF)*, stratified by an alanine aminotransferase (ALT)/quantitative HBsAg (qHBsAg) ratio of 0.2 at the end of treatment (EOT).

For patients with an ALT/qHBsAg ratio <0.2 at EOT, the cumulative incidence of HBsAg seroclearance was significantly higher among patients treated with entecavir than patients treated with TDF (*p* = 0.027, [Fig. 3A]). Multivariate analysis showed that entecavir group, HBV genotype C, NA-experienced status, lower HBV DNA level at initial treatment, lower HBsAg level at EOT and no retreatment were independent factors of HBsAg seroclearance [Supplementary Table 2]. We attempted to find the best cut-off value of ALT/qHBsAg ratio in entecavir and TDF groups. Time-dependent ROC analysis showed that the best cut-off values of ALT/qHBsAg ratio for predicting HBsAg seroclearance within 8 years after cessation of entecavir and TDF treatment were 0.08 and 0.18 (area under the ROC curve: 0.819 and 0.912), respectively. Thus, the optimal values of entecavir and TDF for predicting HBsAg seroclearance were 0.1 and 0.2, respectively. Among patients treated with entecavir, the cumulative rates of HBsAg seroclearance at 8 years after EOT were 56 % and 10.1 % for patients with ALT/qHBsAg ratio ≥0.1 and < 0.1, respectively. Patients with ALT/qHBsAg ratio ≥0.1 exhibited a significantly higher HBsAg seroclearance rate than those with ALT/qHBsAg ratio <0.1 in all patients (*p* < 0.001) and patients with (*p* = 0.043) and without retreatment (*p* < 0.001). Moreover, there was no significant difference in HBsAg seroclearance rates between entecavir groups with ALT/qHBsAg ratio <0.1 and TDF groups with ALT/qHBsAg ratio <0.2 (*p* = 0.126).

### 3.5 Cumulative incidences of HBsAg seroclearance based on qHBsAg of 100 IU/mL at EOT

[Fig. 2] illustrates the cumulative incidences of HBsAg seroclearance for the cohort stratified by a qHBsAg level of <100 IU/mL at EOT. Among patients treated with entecavir [Fig. 2A], the cumulative rates of HBsAg seroclearance at 3, 5, and 8 years after EOT were 23.6 %, 43.4 %, and 58.8 %, respectively, for patients with qHBsAg <100 IU/mL and 1.9 %, 5.0 %, and 13.1 %, respectively, among patients with qHBsAg ≥100 IU/mL. Patients with qHBsAg <100 IU/mL exhibited a significantly higher HBsAg seroclearance rate than patients with qHBsAg ≥100 IU/mL (*p* < 0.001). However, patients with qHBsAg <100 IU/mL exhibited a significantly higher HBsAg seroclearance rate than those with qHBsAg ≥100 IU/mL only in patients without retreatment (*p* < 0.001), but not in patient who received retreatment (*p* = 0.176). Among patients treated with TDF [Fig. 2B], the cumulative rates of HBsAg seroclearance at 3, 5, and 8 years were 25 %, 35.3 %, and 51.2 %, respectively, in patients with qHBsAg <100 IU/mL and 1.2 %, 2.9 %, and 4.1 %, respectively, among patients with qHBsAg ≥100 IU/mL. Patients with qHBsAg <100 IU/mL exhibited a significantly higher HBsAg seroclearance rate than the patients with qHBsAg ≥100 IU/mL (*p* < 0.001), including patients with or without retreatment (both, *p* < 0.001).

**Fig. 2:**
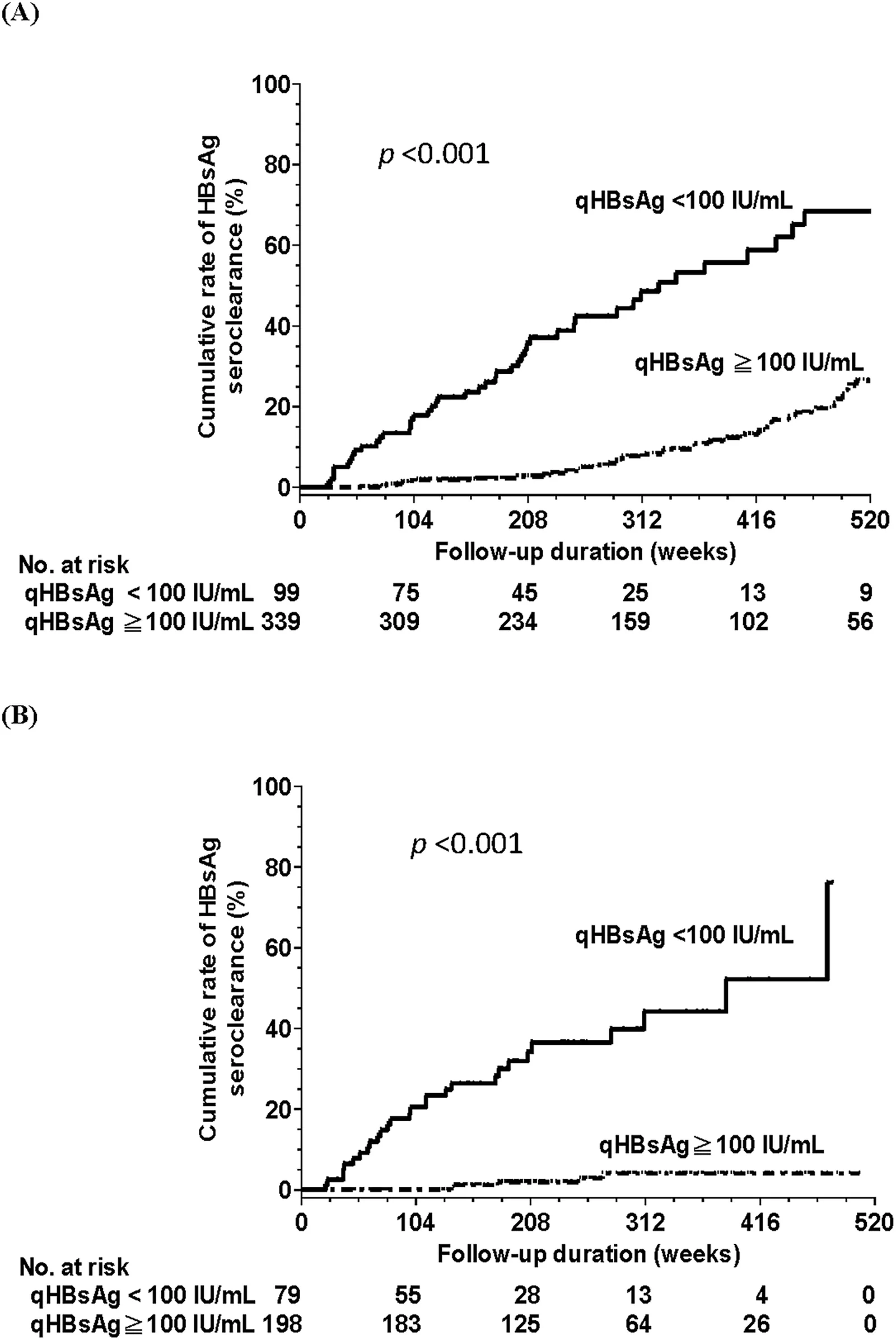
The cumulative incidences of HBsAg seroclearance in patients with HBeAg-negative CHB treated with (A) entecavir or (B) *TDF*, stratified by a qHBsAg level of 100 IU/mL at the end of treatment (EOT).

**Fig. 3:**
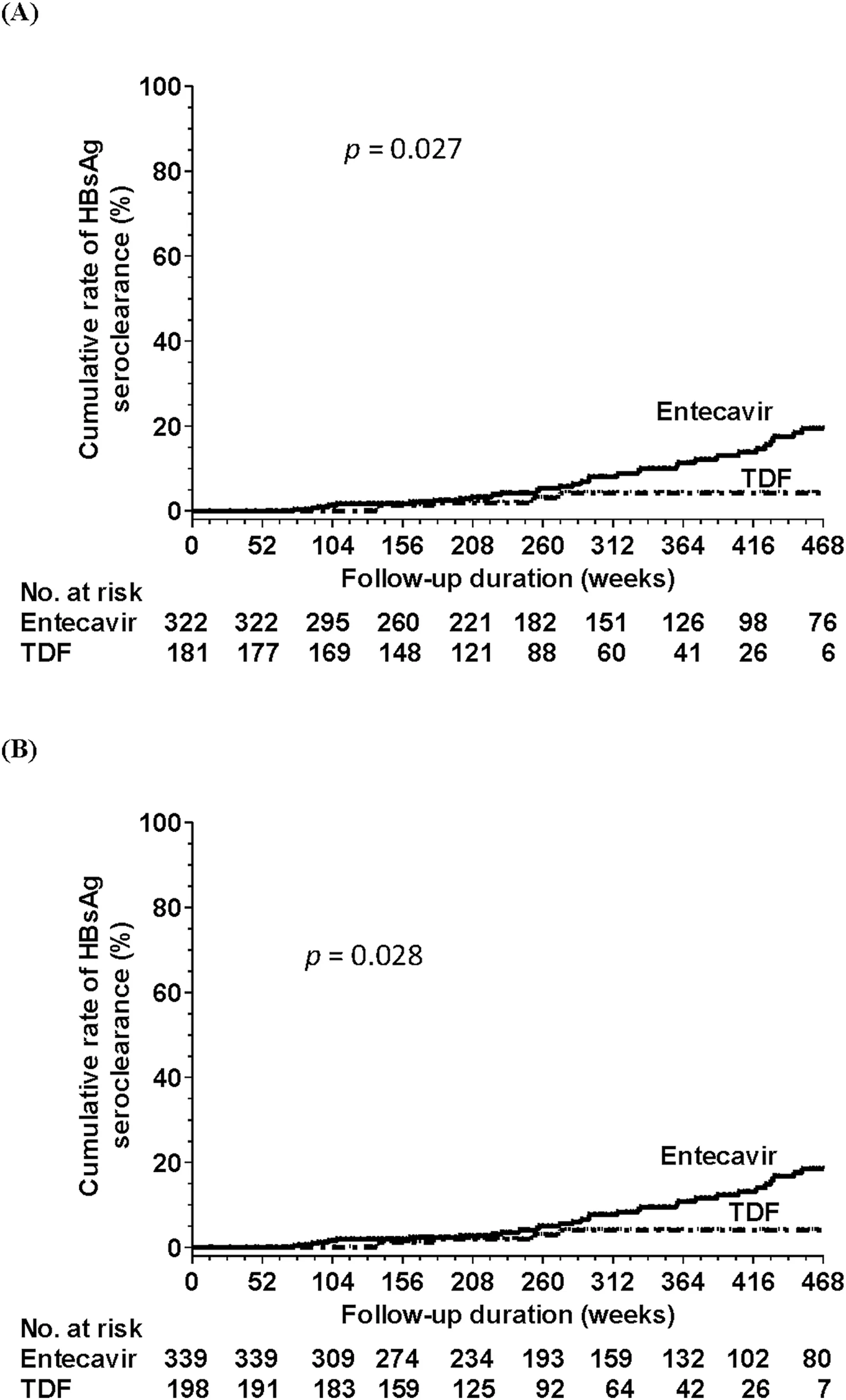
Comparison of the cumulative incidences of HBsAg seroclearance between the entecavir or TDF groups of patients with HBeAg-negative CHB based on (A) a qHBsAg level ≥100 IU/mL and (B) an ALT/qHBsAg ratio ≤0.2 at the end of treatment (EOT).

Among the patients with qHBsAg ≥100 IU/mL at EOT, the cumulative incidence of HBsAg seroclearance was significantly higher among patients treated with entecavir compared to patients treated with TDF (*p* = 0.028, [Fig. 3B]). Multivariate analysis showed that entecavir group, HBV genotype C, NA-experienced status, lower HBsAg level at EOT and no retreatment were independent factors of HBsAg seroclearance [Supplementary Table 2]. We attempted to find the best cut-off values of qHBsAg in entecavir and TDF groups. Time-dependent ROC analysis showed that the best cut-off value of qHBsAg for predicting HBsAg seroclearance within 8 years after cessation of entecavir and TDF treatment were 278.0 and 94.8 IU/mL (area under the ROC curve: 0.801 and 0.919). Thus, the optimal values of entecavir and TDF for predicting HBsAg seroclearance were 300 and 100 IU/mL. Among patients treated with entecavir, the cumulative rates of HBsAg seroclearance at 8 years after EOT were 44.5 % and 8.4 % for patients with qHBsAg <300 IU/mL and ≥300 IU/mL, respectively. Patients with qHBsAg <300 IU/mL exhibited a significantly higher HBsAg seroclearance rate than those with qHBsAg ≥300 IU/mL in all patients (*p* < 0.001) and patients with (*p* = 0.007) and without retreatment (*p* < 0.001). Moreover, there was no significant difference in HBsAg seroclearance rates between entecavir groups with qHBsAg ≥300 IU/mL and TDF groups with qHBsAg ≥100 IU/mL (*p* = 0.269).

### 3.6 Kinetics of the changes in the ALT/qHBsAg ratio and qHBsAg level between EOT and 6 and 12 months post-EOT

Among all patients who achieved HBsAg seroclearance post-treatment, the mean ALT/qHBsAg ratios at EOT and at 6 and 12 months post-EOT were 13.17 ± 47.30, 60.38 ± 187.84, and 106.26 ± 204.67 and the mean qHBsAg levels were 1.59 ± 1.10, 1.25 ± 1.35, and 0.83 ± 1.48 log IU/mL, respectively. Among patients who did not achieve HBsAg seroclearance post-treatment, the mean ALT/qHBsAg ratios at EOT and at 6 and 12 months post-EOT were 0.50 ± 2.89, 4.06 ± 55.34, and 2.81 ± 36.33 and the mean qHBsAg levels were 2.55 ± 0.67, 2.56 ± 1.75, and 2.50 ± 0.76 log IU/mL, respectively [Fig. 4].

**Fig. 4:**
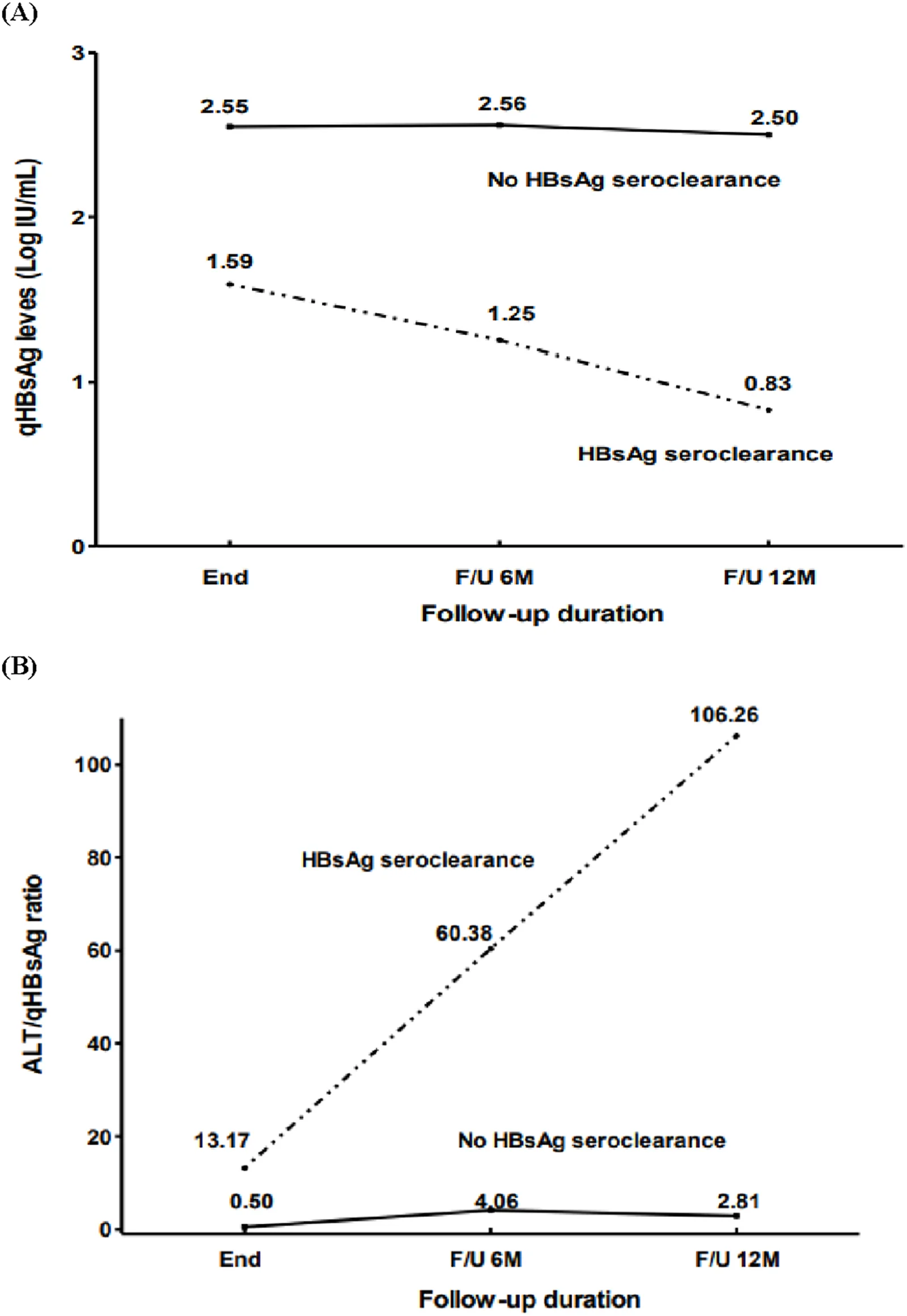
Mean qHBsAg levels and ALT/qHBsAg ratio at different time points (A) qHBsAg levels and (B) ALT/qHBsAg ratio.

In multivariate analysis, the ALT/qHBsAg ratio (model did not include the qHBsAg level at EOT) and qHBsAg level (model did not include the ALT/qHBsAg ratio at EOT) remained independent predictors of subsequent HBsAg seroclearance at 6 and 12 months post-EOT. The HR and 95 % CI for qHBsAg were 0.252 (0.211–0.298, *p* < 0.001) at 6 months and 0.212 (0.176–0.255, *p* < 0.001) at 12 months. The HR and CI for the ALT/qHBsAg ratio were 1.003 (1.002–1.004, *p* < 0.001) at 6 months and 1.005 (1.005–1.006, *p* < 0.001) at 12 months.

Among the patients in the entecavir group who achieved HBsAg seroclearance, the mean ALT/qHBsAg ratios at EOT and at 6 and 12 months post-EOT were 10.42 ± 37.66, 46.71 ± 150.52, and 88.33 ± 197.29 and the mean qHBsAg levels at EOT were 1.76 ± 1.13, 1.51 ± 1.36, and 1.13 ± 1.47 log IU/mL, respectively. For the patients in the entecavir group who did not achieve HBsAg seroclearance, the mean ALT/qHBsAg ratios at EOT and at 6 and 12 months post-EOT were 0.54 ± 3.61, 0.57 ± 3.17, and 1.56 ± 10.08 and the mean qHBsAg levels were 2.57 ± 0.67, 2.58 ± 0.70, and 2.54 ± 0.77 log IU/mL, respectively.

Among the patients in the TDF group who achieved HBsAg seroclearance, the mean ALT/qHBsAg ratios at EOT and 6 and 12 months post-EOT were 20.50 ± 66.77, 96.43 ± 261.67, and 153.54 ± 218.02 and the mean qHBsAg levels were 1.16 ± 0.90, 0.56 ± 1.08, and 0.06 ± 1.19 log IU/mL, respectively. Among the patients in the TDF group who did not achieve HBsAg seroclearance, the mean ALT/qHBsAg ratios at EOT and at 6 and 12 months post-EOT were 0.43 ± 1.23, 8.91 ± 85.23, and 4.62 ± 55.44 and the mean qHBsAg levels at EOT were 2.51 ± 0.66, 2.52 ± 0.81, and 2.45 ± 0.75 log IU/mL, respectively.

For patients who achieved HBsAg seroclearance, TDF was associated with a lower qHBsAg level at EOT (*p* = 0.008) and at 6 months (*p* < 0.001) and 12 months (*p* < 0.001) after EOT compared to entecavir. There were no significant differences in the ALT/qHBsAg ratio at EOT and at 6 or 12 months after EOT between the entecavir and TDF groups.

### 3.7 Comparison of the predictive value of a qHBsAg level and an ALT/qHBsAg ratio at EOT for HBsAg seroclearance

In analysis of the predictive ability for HBsAg seroclearance, a qHBsAg level of 100 IU/mL had significantly lower AIC (1273) and BIC (1276) values than an ALT/qHBsAg ratio of 0.2 (AIC, 1305; BIC, 1308; *p <* 0.001). These results suggest that the qHBsAg of 100 IU/mL has better predictive ability for HBsAg seroclearance than the ALT/qHBsAg ratio of 0.2 at EOT. In additional, a qHBsAg level had significantly lower AIC and BIC values for predicting HBsAg seroclearance than an ALT/qHBsAg ratio at 6 (AIC: 1155 vs. 1362, BIC: 1158 vs. 1365; both *p* < 0.001) and 12 months (AIC: 1068 vs. 1290, BIC: 1071 vs. 1293; both *p* < 0.001) post-EOT.

In addition, a total of 115 HBeAg-negative patients who discontinued entecavir or TDF treatment at China Medical University Hospital were included as a validation cohort. In the analysis of predictive performance for HBsAg seroclearance, a qHBsAg level of 100 IU/mL demonstrated significantly lower AIC (145.77) and BIC (146.82) values than an ALT/qHBsAg ratio of 0.2 at EOT (AIC = 155.96; BIC = 157; *p* < 0.001).

## 4 Discussion

In patients with CHB, achieving a functional cure leads to reduce the liver stiffness and the risk of HCC [21,22]. Therefore, the ability to predict HBsAg loss is crucial during both HBV treatment and after the cessation of NA therapy. In this study, we compared the predictive abilities of an ALT/HBsAg ratio of 0.2 and a HBsAg level of 100 IU/mL at EOT for subsequent HBsAg seroclearance after discontinuation of entecavir or TDF in HBeAg-negative CHB patients. Our findings revealed that, regardless of whether the patients were treated with entecavir or TDF, both an ALT/HBsAg ratio of 0.2 and a HBsAg level of 100 IU/mL at EOT were good predictors of post-treatment HBsAg seroclearance (*p* < 0.001). However, a qHBsAg of 100 IU/mL demonstrated significantly lower AIC and BIC values compared the ALT/qHBsAg ratio of 0.2 at EOT (AIC: 1273 versus 1305, BIC: 1276 versus 1308), which suggests that qHBsAg of 100 IU/mL at EOT is a better predictor of HBsAg seroclearance. Additional, external validation cohort from China Medical University Hospital showed that a qHBsAg level of 100 IU/mL had significantly lower AIC and BIC values than an ALT/qHBsAg ratio of 0.2 at EOT (*p <* 0.001). These results further confirm the role of a qHBsAg level <100 IU/mL in predicting HBsAg seroclearance. Moreover, a qHBsAg level had significantly lower AIC and BIC values for predicting HBsAg seroclearance than an ALT/qHBsAg ratio at 6 and 12 months post-EOT. Therefore, the qHBsAg level may serve as a more reliable predictor of HBsAg seroclearance after discontinuation of entecavir or TDF therapy compared to the ALT/qHBsAg ratio at EOT or during post-treatment follow-up.

A recent study demonstrated that an ALT/qHBsAg ratio of 0.2 could predict HBsAg seroclearance after stopping entecavir treatment. It remained unclear whether the ALT/qHBsAg ratio could accurately predict HBsAg seroclearance after discontinuation of TDF. In our study, both an ALT/qHBsAg ratio of 0.2 and a qHBsAg level of 100 IU/mL at EOT could predict post-treatment HBsAg seroclearance in both the entecavir and TDF groups. However, entecavir was associated with a higher rate of HBsAg seroclearance in patients with an ALT/qHBsAg <0.2 in our cohort (8-year cumulative rate: 13.8 %) compared to the previous study (2.1 %) [12]. In contrast, TDF was associated with a similar HBsAg seroclearance rate in patients with an ALT/qHBsAg <0.2 in our study (8-year cumulative rate: 4.3 %) and the previous study (2.1 %) [12]. Our study found that entecavir was associated with a significantly higher cumulative incidences of HBsAg seroclearance compared to TDF in patients with an ALT/qHBsAg ratio <0.2 and HBsAg level ≥100 IU/mL at EOT. Therefore, entecavir appears to have greater potential to achieve HBsAg seroclearance than TDF in patients with higher HBsAg levels and lower ALT/qHBsAg ratios at EOT. Previous studies have indicated earlier and higher incidences of HBV relapse after discontinuation of TDF therapy compared to entecavir [13,23], and the optimal HBsAg levels for predicting HBV relapse were 150 versus 80 IU/mL for patients treated with entecavir and TDF, respectively [5,13]. In this study, entecavir group, HBV genotype C, NA-experienced status, lower HBsAg level or higher ALT/qHBsAg ratio at EOT and no retreatment were independent factors for HBsAg loss in patients with qHBsAg ≧100 IU/mL or ALT/qHBsAg ratio <0.2 at EOT. In these factors, only HBV DNA levels at initial treatment were lower in entecavir group compared with TDF group. In addition, entecavir group had the higher cumulative incidences of virological and clinical relapse and retreatment than TDF group (*p* < 0.05) in patients with qHBsAg ≧100 IU/mL or ALT/qHBsAg ratio <0.2 at EOT, but not in patients with qHBsAg <100 IU/mL or ALT/qHBsAg ratio ≧0.2 at EOT. We thick these may be the reasons for the different HBsAg seroclearance rates between the entecavir and TDF groups in patients with qHBsAg ≧100 IU/mL or ALT/qHBsAg ratio <0.2 at EOT. One Previous meta-analysis study showed that TDF and entecavir were equally effective in the treatment of patients with NA-naive CHB [24]. Therefore, drug potency is unlikely to be the reason for the different clinical outcomes observed after treatment cessation between entecavir and TDF therapy. However, we still do not fully understand the underlying mechanism.

Therefore, establishing optimal values of qHBsAg and the ALT/HBsAg ratio at EOT is necessary to predict HBsAg seroclearance for patients who discontinued entecavir and TDF therapy. In our study, among patients treated with entecavir, the optimal values of qHBsAg and the ALT/HBsAg ratio at EOT were 300 IU/mL and 0.1, respectively. In contrast, for patients treated with TDF, the optimal values at EOT were 100 IU/mL for qHBsAg and 0.2 for the ALT/HBsAg ratio. These optimal values were able to predict HBsAg seroclearance, including in patients with or without retreatment, in both the entecavir and TDF groups.

In this study, the patients with an ALT/qHBsAg <0.2 in the entecavir group appeared to have a higher HBsAg seroclearance rate compared to the previous study [12]. In the previous study, patients resumed entecavir treatment if virological relapse occurred; in the current study, retreatment was based on clinical relapse. Previous studies have shown that remaining off-treatment is associated with a higher rate of HBsAg loss compared to retreatment, even among patients who experience clinical relapse [8,25]. ‘Host-dominating flares' indicate the presence of an effective immune response. Such flares may resolve with ALT normalization and a decline HBsAg levels, and thus increase the rate of HBsAg seroclearance [26].

We also investigated the kinetics of the changes in the qHBsAg levels and ALT/qHBsAg ratio between EOT and 6 and 12 months post-EOT. The qHBsAg levels and ALT/qHBsAg ratio did not change significantly after EOT in patients who did not achieve seroclearance. However, in patients who achieved seroclearance, the qHBsAg levels gradually decreased and the ALT/qHBsAg ratio gradually increased after EOT. Therefore, analysis of both the absolute values and kinetic changes in qHBsAg and the ALT/qHBsAg ratio for up to 12 months after EOT may help to predict HBsAg seroclearance. In addition, among the patients who achieved seroclearance, TDF was associated with a lower qHBsAg level at EOT and 6 and 12 months post-EOT than entecavir. Therefore, patients treated with TDF may need to achieve lower qHBsAg levels at EOT in order to achieve subsequent HBsAg seroclearance.

There are some limitations to this study. First, this retrospective study was conducted at a single referral center; thus, further multicenter and prospective studies are needed. Second, we only included Asian patients and the predominant HBV genotypes in our cohort were B and C. Therefore, it is unclear whether the different predictive value of a qHBsAg level or an ALT/qHBsAg ratio after cessation of entecavir or TDF can be generalized to other populations or HBV genotypes.

In conclusion, a qHBsAg level of 100 IU/mL at EOT demonstrates better predictive value for HBsAg seroclearance compared to an ALT/qHBsAg ratio of 0.2 after cessation of entecavir or TDF in HBeAg-negative CHB patients. Furthermore, the qHBsAg levels at EOT and 6 and 12 months post-EOT may be useful tools for predicting HBsAg seroclearance. The optimal values for predicting HBsAg seroclearance were 300 and 100 IU/mL for qHBsAg and 0.1 and 0.2 for the ALT/qHBsAg ratio after cessation of entecavir versus TDF, respectively. Therefore, entecavir appears to have a higher potential for achieving HBsAg seroclearance than TDF in patients with higher HBsAg levels or lower ALT/qHBsAg ratio at EOT.

## Funding

*This work was* supported *by* the 10.13039/100007225Ministry of Science and Technology *(*Grant *number* NMRPG8L6121*)* and 10.13039/100012553Chang Gung Memorial Hospital, Taiwan *(*Grant *number* CMRPG8N0981).

## A conflict of interest declaration

Cheng-Yuan Peng has served as an advisory committee member for AbbVie, BMS, Gilead, and MSD. There is no conflict of interest to disclose for other authors.

## Figures and Tables

**figs1:**