# Supplementary material for: qHBsAg outperforms ALT/qHBsAg ratio in predicting HBsAg seroclearance after cessation of entecavir versus tenofovir
Source: Biomed J. 2026 Jan 13;49(5):100945. doi: 10.1016/j.bj.2026.100945 (PMC13625803; doi:10.1016/j.bj.2026.100945)
Supplement: Supplementary file 1 [file mmc1.docx]

**Supplementary Table 1.** The definitions and incidences of virological and clinical relapse, retreatment and HBsAg seroclearanc after entecavir or TDF discontinuation

| Outcome Events | Definition | The incidences of HBV relapse, retreatment and HBsAg seroclearance at 3, 5 and 10 years after cessation of entecavir or TDF therapy |
| --- | --- | --- |
| Virological relapse | Serum HBV DNA level >2000 IU/mL after the cessation of NA treatment | 74.4%, 76.9% and 79.4%, respectively. |
| Clinical relapse | Serum ALT elevation > 2 × ULN (40 U/L) plus HBV DNA >2000 IU/mL after stopping NA treatment | 56.2%, 60.3% and 63.2%, respectively |
| Retreatment | Patients received antiviral agents after HBV relapse if they met the criteria of Taiwan's National Health Plan. | 44%, 50.9% and 56.4%, respectively |
| HBsAg seroclearance | Loss of HBsAg in the serum, confirmed by at least one subsequent negative HBsAg test, with or without the development of hepatitis B surface antibody (anti-HBs). | 7.3%, 13% and 33.8%, respectively. |

ALT, alanine aminotransferase; HBV, hepatitis B virus, HBsAg: hepatitis B surface antigen; NA, nucleoside analogues; TDF, tenofovir disoproxil fumarate.

**Supplementary Table 2.** Factors predictive of HBsAg seroclearance for patients with qHBsAg≧100 IU/mL (Model 1) or ALT/qHBsAg ratio <0.2 (model 2) by multivariate analysis

| Variables | *Multivariate analysis (model 1) | | *Multivariate analysis (model 2) | |
| --- | --- | --- | --- | --- |
|  | HR (95% CI) | *P* value | HR (95% CI) | *P* value |
| Initial treatment |  |  |  |  |
| TDF vs. entecavir | 0.272 (0.103-0.716) | 0.008 | 0.323 (0.119-0870) | 0.025 |
| HBV DNA (per log IU/mL) |  |  | 0.755 (0.612-0.930) | 0.008 |
| HBV genotype (C vs. B) | 3.121 (1.616-6.029) | 0.001 | 3.296 (1.601-6.786) | 0.001 |
| NA-naïve (yes vs. no) | 0.484 (0.259-0.903) | 0.023 | 0.412 (0.216-0.787) | 0.007 |
| End of treatment |  |  |  |  |
| qHBsAg (per log IU/mL) | 0.164 (0.076-0.356) | <0.001 | N/A |  |
| ALT/qHBsAg ratio | N/A |  | 2.064 (1.067-3.082) | <0.001 |
| Retreatment (yes vs. no) | 0.283 (0.147-0.542) | <0.001 | 0.318 (0.166-0.611) | 0.001 |

*Model 1: Multivariate analysis included qHBsAg, no ALT/qHBsAg at EOT. *Model 2: Multivariate analysis included ALT/qHBsAg, no qHBsAg at EOT.

ALT, alanine aminotransferase; CI, confidence interval; HR, Hazard ratio; HBV, hepatitis B virus, HBsAg: hepatitis B surface antigen; NA, nucleoside analogues; N/A: Not applicable; qHBsAg (quantitative HBsAg); TDF, tenofovir disoproxil fumarate.
